# SEMA4A promotes prostate cancer invasion: involvement of tumor microenvironment

**DOI:** 10.7150/jca.86739

**Published:** 2023-08-21

**Authors:** Xiao Liu, Weiwei Tan, Weiqi Wang, Tingting Feng, Chunni Wang, Lin Wang, Wei Zhou

**Affiliations:** 1 Department of Oncology, Zibo Central Hospital, Zibo, China.; 2 Department of Pathology, Huazhong University of Science and Technology Union Shenzhen Hospital, Shenzhen, China.; 3 Institute of Radiation Medicine, Shandong First Medical University, Shandong Academy of Medical Sciences, Jinan, China.; 4 The Key Laboratory of Experimental Teratology, Ministry of Education and Department of Pathology, School of Basic Medical Sciences, Shandong University, Jinan, China.; 5 Department of Radiation Oncology, Shandong Cancer Hospital and Institute, Shandong First Medical University and Shandong Academy of Medical Sciences, Jinan, China.; 6 Biomedical Sciences College & Shandong Medicinal Biotechnology Centre, NHC Key Laboratory of Biotechnology Drugs, Key Lab for Rare & Uncommon Diseases of Shandong Province, Shandong First Medical University & Shandong Academy of Medical Sciences, Jinan, China.; 7 Department of Radiation Oncology, Qilu Hospital, Cheeloo College of Medicine, Shandong University, Jinan, China.

**Keywords:** prostate cancer, SEMA4A (Semaphorin 4A), IL-10 (Interleukin-10), epithelial-mesenchymal transition, invasion, stromal

## Abstract

Semaphorin 4A (SEMA4A) belonged to a family of membrane-bound proteins that were initially recognized as a kind of axon guidance factors in nervous system. It was preferentially expressed on immune cells and has been proven to play a prominent role in immune function and angiogenesis. In this study, we found that SEMA4A was highly expressed in prostate cancer (PCa) tissues and correlated with Gleason scores and distant metastasis. SEMA4A could induce Epithelial-mesenchymal transition (EMT) of PCa cells and consequently promote invasion by establishing a positive loop with IL-10 in stromal cells. *In vivo* experiments showed more dissemination in mice injected with SEMA4A-overexpressing cells in mouse models and both the number and size of lung metastases were significantly increased in SEMA4A-overexpressing tumors. SEMA4A depletion by genetic means prevents lung metastasis in PCa xenograft models. Our data suggest a crucial role of SEMA4A in PCa and blocking SEMA4A-IL-10 axis represents an attractive approach to improving therapeutic outcomes.

## Introduction

Prostate cancer (PCa) is a heterogeneous disease with high incidence and mortality^1^, ^2^, and metastasis is a significant risk factor for the mortality^3^. Cancer metastasis consists of multiple biological progresses like angiogenesis, migration and invasion of tumor cells, and tumor-stromal interaction in the hostile microenvironment^4^. Therefore, identifying the key factors and its pathological mechanism that promotes the metastasis of PCa could provide novel treatment strategy.

Semaphorins are a family of secreted and membrane-bound proteins that were initially recognized as a kind of axon guidance factors in nervous system and were correlated with development of organs like heart, kidney and immune system^5^. Currently, increasing evidence indicated that they were deregulated in patients with carcinomas^6^ and might affect the apoptosis, migration and immune escape of tumor cells^7^. However, numerous studies also demonstrated that its family members functioned in a tissue-specific manner^8^. Semaphorin 4A (SEMA4A) belongs to the family of Semaphorins, which is composed of 761 amino acids^9^. It exists primarily as membrane-bound protein, but its extracellular portion can be processed into soluble form by proteolytic cleavage^7^. SEMA4A was preferentially expressed on antigen-presenting cells (APC) such as dendritic cells (DC) and B cells, and involved in immune responses by regulating T cells^10^. However, its expression and function in PCa cells remains to be characterized.

In this study, we found that SEMA4A was highly expressed in PCa tissues and the upregulation of of SEMA4A expression in PCa cells was closely related to the invasive phenotype of PCa cells. In addition, SEMA4A could regulate the secretion of IL-10 in stromal cells and coordinates with IL-10 to promote tumor cell invasion via inducing Epithelial-mesenchymal transition. This SEMA4A-IL-10 axis may represent an important pathway in the interaction between PCa and stromal cells and hold great promise for therapeutic intervention in PCa.

## Materials and methods

### Tissue collection

The tumor samples were obtained from Qilu Hospital of Shandong University (Jinan, China) and The Affiliated Hospital of Qingdao University (Qingdao, China) between 2001 and 2012. All of these patients were hospitalized due to symptoms of lower tract urinary obstruction. None of the patients received preoperative radiation or androgen deprivation therapy. Besides, none of them were diagnosed as rheumatoid arthritis, inflammatory bowel disease or multiple sclerosis that may significantly impact the expression status of SEMA4A and prognosis of the enrolled patients. This study was conducted in accordance with the International Ethical Guidelines for Biomedical Research Involving Human Subjects. This study protocol was approved by Shandong Medicinal Biotechnology Centre Ethics Committee according to the Declaration of Helsinki (Document No. SMBC17LL017). The informed written consent was obtained from the patients.

### Immunohistochemistry (IHC)

IHC was performed as previously described^11^. Briefly, the sections were incubated with primary antibody at 4°C overnight, then were blindly evaluated by two independent observers (T.F and L.W.), and the following scoring system was utilized to validate SEMA4A expression. The cytoplasmic staining was scored into four grades, which were classified by its staining intensity: 0, 1+, 2+, and 3+. The percentages of SEMA4A-positive cells were scored into five categories: 0 (0%), 1(1-25%), 2(26-50%), 3(51-75%), and 4 (76-100%). A final score was built by multiplying the scores of these two parameters, which are defined as follows: 0, negative; 1-3, weak; 4- 6, moderate; and 8-12, strong.

### Cells culture and treatment

Four human prostate cancer cell lines 22RV1, PC-3, LNCaP and VCaP as well as a human prostatic stromal myofibroblast cell line WPMY-1 were obtained from American Type Culture Collection (ATCC, Manassas, VA, USA), and cultured as the instructions. Cells were seeded in 2%FBS medium at least 16 hours before drugs addition and then were given one or some of the following treatments for 72 hours. (**I**) recombinant human SEMA4A (rhSEMA4A, 10 or 100 ng/ml, Abnova); (**II**) IL-10 (0.1, 1, 10 or 100 ng/ml, Peprotech); (**III**) IL-10 neutralizing antibody (5 μg/ml, Peprotech); STAT3 inhibitor stattic (10μM, Sigma-Aldrich). Medium containing above drugs or corresponding vehicles was changed every 24 hours. The dose and timing were chosen based on preliminary experiments showing optimal gene expressions.

### RNA extraction, reverse-transcription PCR (RT-PCR) and qRT-PCR analysis

RNA extraction, RT-PCR and qRT-PCR were performed as previously described^11^. Relative mRNA levels were measured using 2^-△△CT^ method. Three independent experiments were conducted and each reaction was in triplicate. The details of the primers for each gene were listed in Supplementary Table 1. GAPDH was used as loading control.

### Chromatin Immunoprecipitation (ChIP) Assay

22RV1 cells were stimulated with IL-10 ng/ml for 24 hr or left unstimulated. Chromatin from 22RV1 cells was fixed and immunoprecipitated using the ChIP assay kit as recommended by the manufacturer (Beyotime Biotechnology, China). The purified chromatin was immunoprecipitated using 2 mg of anti-STAT3 (ab267373, Abcam) or irrelevant antibody (anti-IgG, Santa Cruz). After DNA purification, the presence of the selected DNA sequence was assessed by PCR and qRT-PCR and the primers were as followings: forward, 5'-CCCAGCCCCCTGACCAAATTTTA-3'; reverse, 5'-AAGAAAGAGTGGCTGGGAGGTT-3'.

### siRNA mediated gene silence

The detail of siRNA transfection on prostatic cells was described before^11^. Briefly, cells were transiently transfected with Sema4A small interfering RNA (siRNA, 50 nM, SI00133560, SI04174107, QIAGEN, Hilden, Germany) or negative control (QIAGEN, SI03650318) by Hiperfect transfection reagent (QIAGEN) following the manufacturer's instructions, and all experiments were performed 24-48 h after transfection.

### Transwell assay

Transwell assay was performed as previously described^12^. The experiments were performed with triplicates and in three times.

### Western blot

Western blot was performed as previously described^12^. Briefly, the membrane was incubated overnight at 4°C with primary antibody against SEMA4A (1:500, Proteintech), E-cadherin (1:1000, Cell Signaling Technology), ZO-1 (1:1000, Cell Signaling Technology), STAT3 (ab267373, Abcam), STAT3 (ab76315, Abcam) or Vimentin (1:1000, Cell Signaling Technology), GAPDH (1:1000, Santa Cruz) was used as loading control. Three independent experiments were performed.

### Cytokine array analysis

Cytokine array analysis was performed by using the Human Cytokine Array Panel A Array Kit (R&D Systems). The culture supernatant was collected and then examined according to the protocol.

### Enzyme-linked immunosorbent assay (ELISA)

The secretion of SEMA4A or IL-10 was detected by ELISA kit according to the instructions of the manufacturer, respectively. The human IL-10 ELISA kit was from R&D system and the human SEMA4A ELISA kit was from CUSBIO.

### Indirect co-culture of PCa cells and stromal cells

5000 WPMY-1 cells were plated into flat bottom 24-well plates, and 5000 22RV1 cells were seeded into the inserts with a 0.4 μm pore size (Corning). Both could adhere overnight. Then the inserts were placed in appropriate 24-well plates and were given the treatments of IL-10 neutralizing antibody, siSEMA4A or stattic, and then incubated for 6 days.

### Mice models

The experimental protocols were approved by the Institutional Animal Care and Use Committee of Shandong Medicinal Biotechnology Centre (Document No. 2017LL032). The investigation conformed to the US National Institutes of Health Guide for the Care and Use of Laboratory Animals and was performed in accordance with the ARRIVE guidelines (http://www.nc3rs.org/ARRIVE). All mice were housed under specific pathogen-free conditions and maintained on a 12-h light/dark cycle at 25±2°C, with free access to food and water. Six-week-old female BALB/c nude mice (nu/nu) were purchased from Vital River Laboratories (Bejing) and acclimated to housing conditions for at least 1 week before experiments. We subcutaneously injected mice with VCAP cells with SEMA4A overexpression (1×10^7^) or not into nude mice alone or together with WPMY-1 cells (1×10^5^), then on days 6 after injection, cell-implanted mice implanted were divided into 6 groups (n=8 each) for intraperitoneal injection of rhSema4A (10 mg/kg body weight), stattic (5 mg/kg body weight), PBS or DMSO every 3 days. Metastatic lungs were fixed in picric acid for quantifying the number of metastasis loci. The formalin (10%) fixed tissue samples were embedded in paraffin, and then hematoxylin and eosin (H&E) staining was performed according to standard protocol. The H&E-stained slides were observed with a model BH2 microscope (Olympus Corp., Tokyo, Japan).

### Statistics

Statistical analysis was performed using the Statistical Package for Social Sciences, version 19.0 (SPSS). P<0.05 was considered statistically significant.

## Results

### Expression of SEMA4A in PCa tissues and its clinicopathological significance

**Figure 1:**
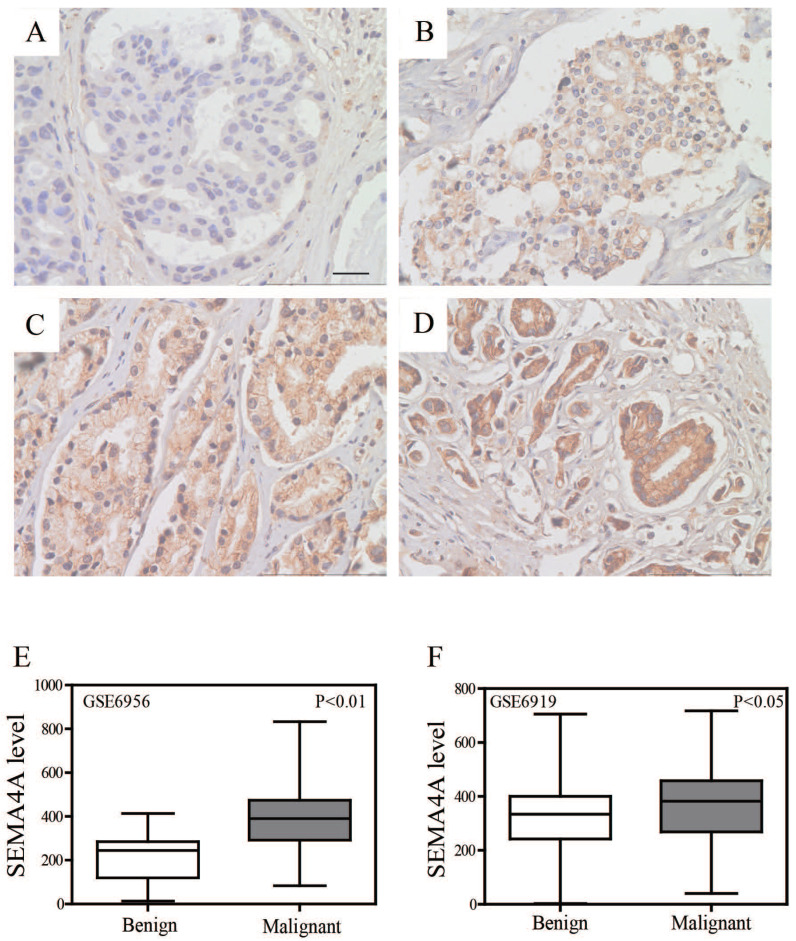
** Expression of SEMA4A in PCa tissues.** Representative IHC images of SEMA4A were shown (original magnification, ×400). (A) Negative staining of SEMA4A. (B) Weak-positive staining of SEMA4A. (C) Moderate-positive staining of SEMA4A. (D) Strong-staining of SEMA4A. (E and F) SEMA4A expression in public datasets (GSE6956 and GSE6919) was comparatively plotted in benign and malignant prostatic tissues.

The protein levels of SEMA4A were analyzed by immunohistochemistry (IHC) in a series of 93 PCa and 12 benign prostatic hyperplasia (BPH) tissues. Overall, its expression level was significantly higher when compared with adjacent non-tumor tissues and BPH ones. For the analysis, we grouped the PCa samples into negative-weak and moderate-strong categories. SEMA4A was moderately or strongly positive in 30 of the 93 interpretable cases (32%) with PCa but in 3 of the 12 interpretable cases (25%) with BPH. Representative IHC images of SEMA4A in PCa are shown in Figure 1A-D.

Concerning the cellular locations of SEMA4A, positive staining was confined mainly to the nucleus and cytoplasm compared to a negatively stained adjacent non-tumor tissue and BPH ones. However, no visible labeling was detected in the stroma. Consistently, we re-analyzed two public datasets (GSE6956 and GSE6919) and found that SEMA4A was significantly higher expressed in malignant tissues when compared with benign ones (Figure 1E and F).

To determine the clinical significance of SEMA4A in PCa, the relationship between SEMA4A expression and clinicopathological characteristics was analyzed. We found that high-expression of SEMA4A was significantly correlated with preoperative PSA (P<0.001), Gleason score (P<0.001) and distant metastasis (P=0.035) (Table 1). It suggested that SEMA4A might be associated with pathological grades and metastasis of PCa.

### SEMA4A promotes migration and invasion of PCa cells* in vitro*

**Figure 2:**
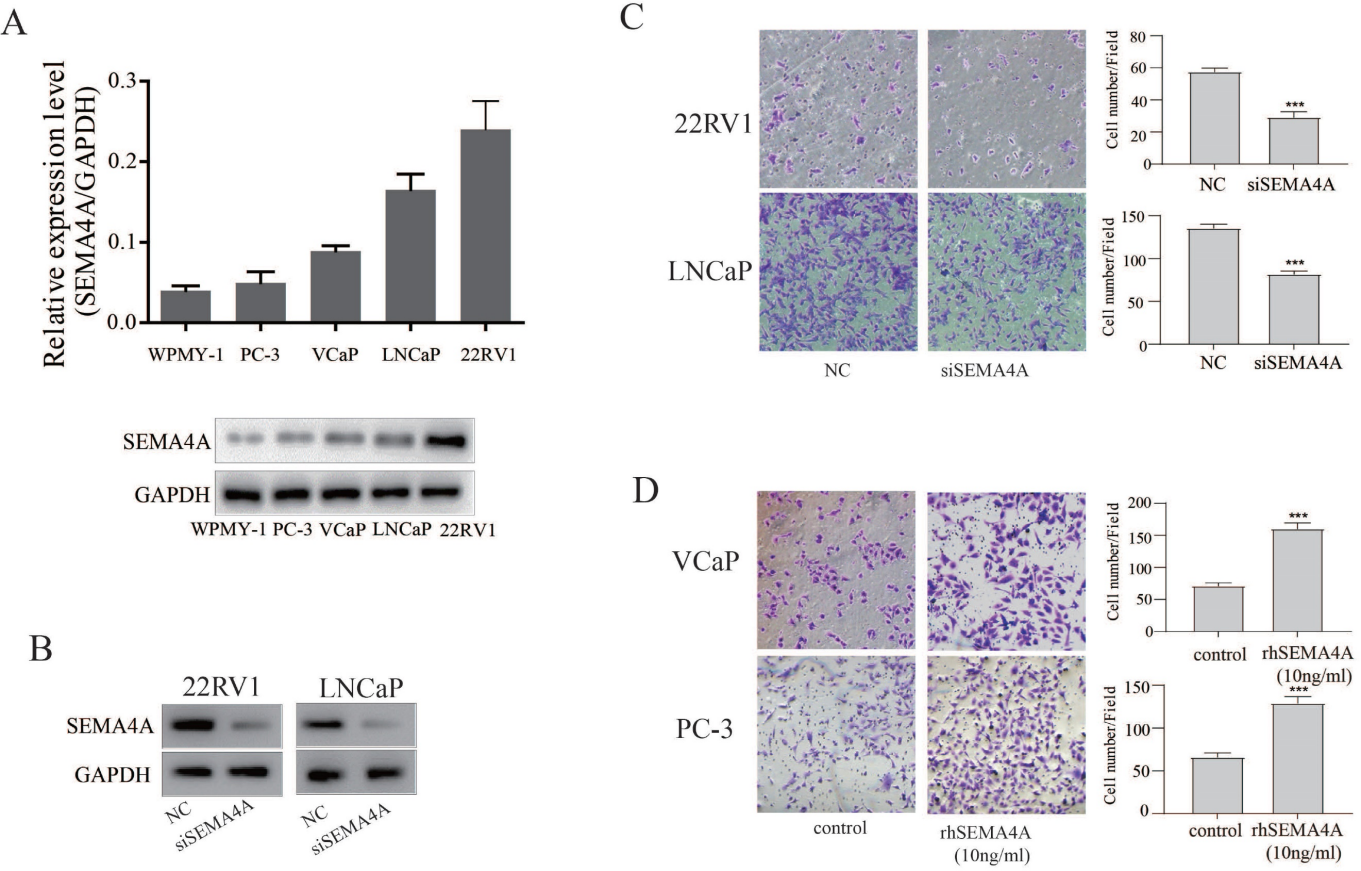
** SEMA4A promoted migration* in vitro*.** (A) The expression of SEMA4A in PCa cell lines was detected by RT-qPCR (upper panel) and Western blot (lower panel). (B) The silencing efficiency of siRNA targeting SEMA4A was detected by Western blot. (C) The migration of 22RV1 and LNCaP cells was measured when SEMA4A was silenced. (D) The migration was detected when VCaP and PC-3 cells were challenged by human recombinant SEMA4A (rhSEMA4A). *P<0.05, **P<0.01.

To investigate the biological function of SEMA4A in PCa, we firstly detected the basal expression levels of SEMA4A in various PCa cells and stromal cells. As shown in the Figure 2A, SEMA4A was abundantly expressed in PCa cells, but not in stromal cells (WPMY-1). Among them, its expression was highest in 22RV1 but lowest in PC-3 cells. SiRNA targeting SEMA4A was further applied, which could suppress its expression efficiently in 22RV1 and LNCaP (Figure 2B). Then, we examined the biological function of endogenous SEMA4A and the transwell assay showed that silencing SEMA4A significantly suppressed the migration of 22RV1 and LNCaP cells (Figure 2C) whereas rhSEMA4A treatment could promote the migration of VCaP and PC3 (Figure 2D). However, no visible effect on the proliferation was detected in PCa cells (data not shown).

**Figure 3:**
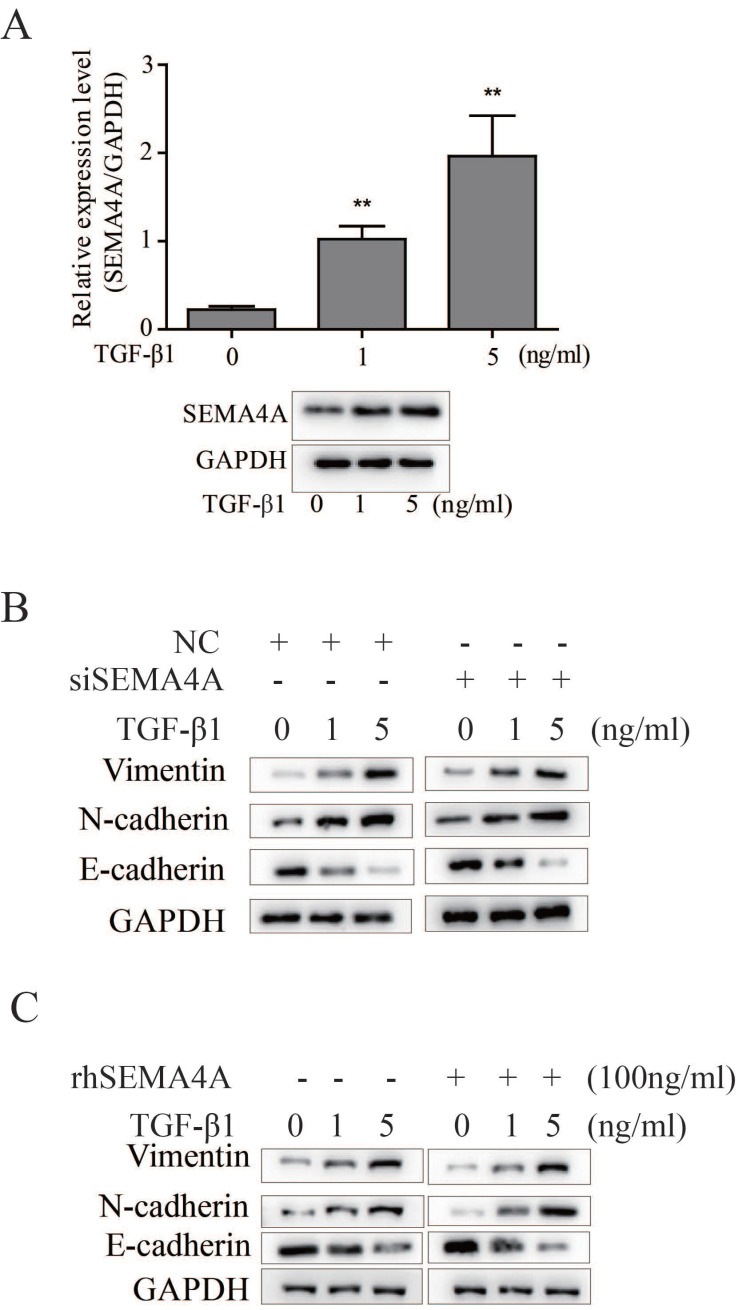
** SEMA4A promoted EMT* in vitro*.** (A) TGF-β1 increased SEMA4A expression at transcript (upper panel) and protein (lower panel) levels in 22RV1 cells. (B) The effects of SEMA4A knockdown on the TGF-β1-induced EMT. (C) rhSEMA4A treatment further promoted TGF-β1-induced EMT. *P<0.05, **P<0.01.

Epithelial-mesenchymal transition (EMT) is a critical event during PCa metastasis^13^ and previous studies have demonstrated that SEMA4A confers doxorubicin resistance on hepatocellular carcinoma by inducing epithelial-mesenchymal transition (EMT)^14^. As described before, TGF-β1 is a typical inducer of EMT during PCa progression^11^. In this study, we further evaluated the potential role of SEMA4A on TGF-β1-induced EMT. As shown in Figure 3A, TGF-β1 can induces SEMA4A expression in 22RV1 cells, importantly, detailed study demonstrated that silencing SEMA4A could attenuate the TGF-β1-induced EMT, as evidenced by the restoration of epithelial marker, such as E-cadherin, but the blockade of the induction of mesenchymal markers (Vimentin and N-cadherin) (Figure 3B). Following study showed that rhSEMA4A treatment exerted a duplicate effect on the EMT induction in the presence of TGF-β1, as demonstrated by the further increase of Vimentin and N-cadherin, but the more obvious decrease of E-cadherin (Figure 3C). Notably, silencing SEMA4A expression or rhSEMA4A treatment alone could sufficiently inhibit or promote EMT (Figure 3B and 3C), which indicates the existence of a TGF-β1-independent way during SEMA4A functioned.

### SEMA4A requires stromal cells to induce the metastatic phenotype of PCa

**Figure 4:**
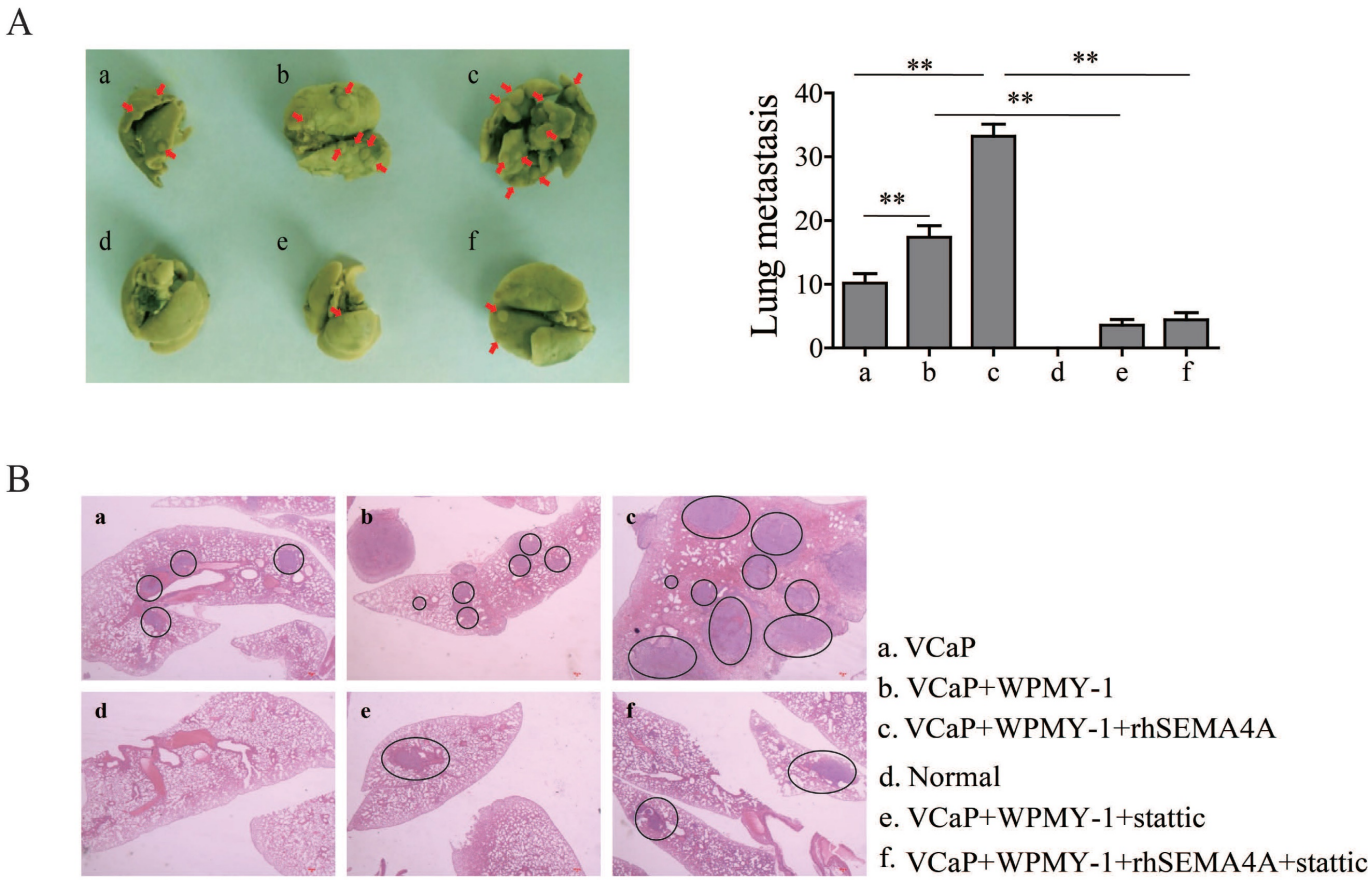
** SEMA4A requires stromal cells to induce the metastatic phenotype of PCa.** (A) rhSEMA4A increased the metastatic foci on the lung, but stattic decreased the foci* in vivo* experiments. The red arrows indicated the metastatic foci. (B) Representative HE staining images of the foci were shown. The black circle indicates the metastatic foci. **P<0.01.

It is widely accepted that the tumor microenvironment, or stromal compartment, is biologically heterogeneous and the interactions of its components with cancer cells is required for metastasis of cancer cells to ectopic sites. Among the various cell types located in the tumor microenvironment, stromal cells could promote tumor growth and metastasis in PCa^15^. To confirm the role of SEMA4A in the pro-metastatic effects of environment involving PCa and stromal cells, a xenograft tumor-bearing model was established by inoculating VCaP cells with SEMA4A overexpression or not into nude mice alone or together with WPMY-1. As expected in Figure 4A, more metastases foci were observed when VCaP with SEMA4A overexpression and WPMY-1 were inoculated simultaneously than their parental controls. Importantly, when the nude mice were intra-tumoral injected with rhSEMA4A, the metastases foci increased dramatically when compared with the control ones injected with vehicle. The results further support that SEMA4A is a functional element in the tumor microenvironment to exacerbate disease progression. Representative HE staining images are shown in Figure 4B. In contrast, knockdown of SEMA4A significantly reduced mammary tumor metastasis in the lungs.

### SEMA4A and IL-10 are involved in the interaction between PCa and stromal cells

**Figure 5:**
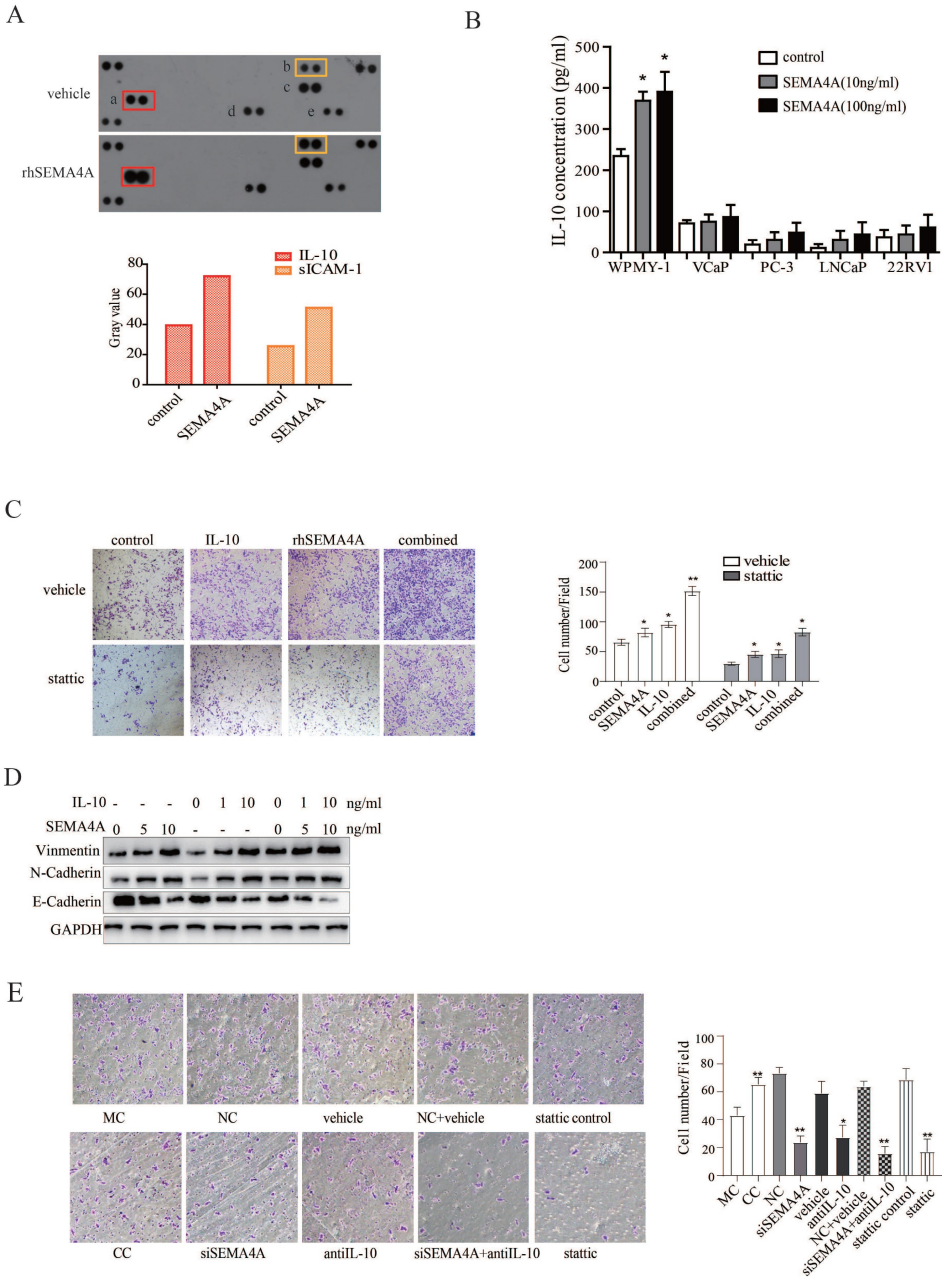
** SEMA4A and IL-10 are involved in the interaction between PCa and stromal cells.** (A) rhSEMA4A up-regulates the expression of IL-10 and sICAM-1in WMPY-1. (B) rhSEMA4A treatment up-regulated secretion of IL-10 in WMPY-1. (C) IL-10 and rhSEMA4A alone or combined treatment could promote migration of VCaP, and stattic (10μM), a selective STAT3 inhibitor, could block the phenotype. (D) IL-10 and SEMA4A synergistically promote EMT. (E) Co-culture of 22RV1 and WPMY-1 enhanced the migration of 22RV1, and the ability was attenuated when SEMA4A, IL-10 or STAT3 signaling was silenced or blocked. *P<0.05, **P<0.01.

To further study the biological function of SEMA4A in tumor microenvironment, a human cytokine array was utilized to test its effect on cytokine production of WPMY-1, a stromal cell line. As shown in Figure 5A, the results revealed that, after rhSEMA4A treatment, the production of IL-10 (a) and sICAM-1 (b) increased significantly when compared with its vehicle control, although MIF (d) and Serpin E1 (e) were also up-regulated, but not reach statistical significance.

As the expression and function of sICAM-1 in cancer has been well characterized, which further supports the pro-migration ability of SEMA4A in the pathological progression of PCa, more attention is paid on the biological activity of IL-10, as well as its relation with SEMA4A. Next, we performed an ELISA assay and the data further verified that stimulation with rhSEMA4A significantly induced the secretion of IL-10 in the supernatant of WPMY-1 (Figure 5B).

Previous study reported that IL-10 could promote tumorigenesis or progression through STAT3 signaling in diverse diseases, like chronic lymphocytic leukemia and breast cancer^16^. Furthermore, IL-10 produced by M2-polarized tumor-associated macrophages was related with EMT in pancreatic cancer^17^. To explore the biological function of IL-10, we performed a transwell assay and observed that IL-10 could promote the migration capability of PCa cells (Figure 5C). Interestingly, combined treatment with rhSEMA4A and IL-10 showed a duplicate effect towards the migration of PCa cells as well as the occurrence of EMT when compared with the one alone (Figure 5D), suggesting the existence of cooperation between IL-10 and SEMA4A to exacerbate the progression of PCa.

As cytokines have been reported to be involved in the interaction between PCa with the stromal compartment, we speculated that the IL-10 secretion by SEMA4A stimulation may be involved in this interaction to promote invasion of PCa cells. To confirm this hypothesis, indirectly co-culture method was applied. As expected, after co-cultured 22RV1 with WPMY-1 for 6 days, the migration ability of 22RV1 cells was up-regulated (Figure 5E), suggesting the existence of some factors was functional for this. To further confirm this conclusion, siSEMA4A and IL-10 neutralizing antibody were applied and, siSEMA4A or IL-10 neutralizing antibody treatment alone could inhibit the migration of 22RV1 when co-cultured with WPMY-1(Figure 5E). Importantly, the combined treatment with siSEMA4A and IL-10 antibody could almost abolish the pro-migratory effects of WPMY-1 on 22RV1 (Figure 5E). These results indicated the existence of a positive feed-back loop involving SEMA4A and IL-10 in the tumor microenvironment to promote PCa progression.

### STAT3 serves as a critical factor in the SEMA4A-IL-10 feed-back mechanism

**Figure 6:**
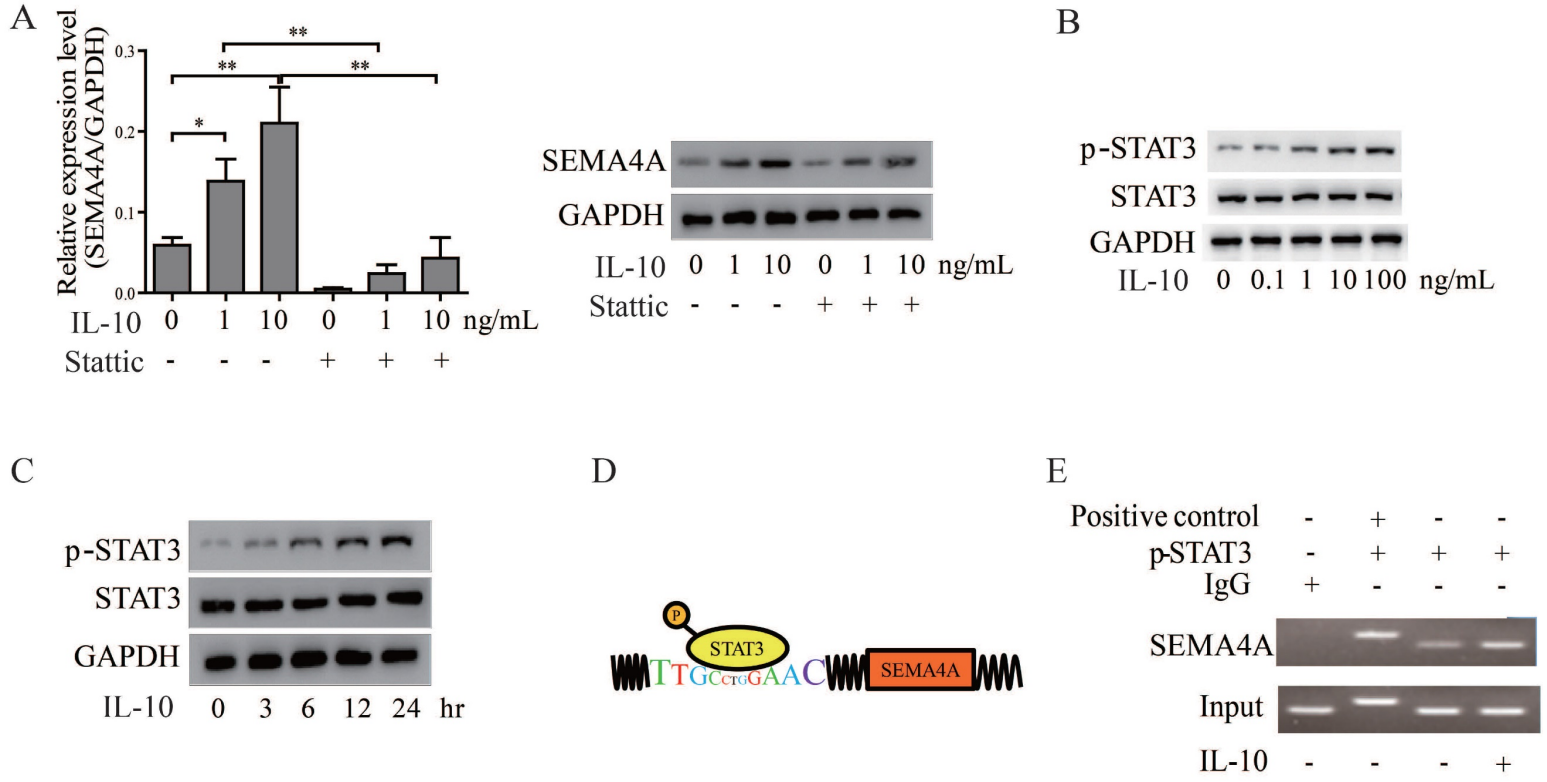
** SEMA4A was directly regulated by STAT3.** (A) IL-10 increased SEMA4A expression at transcript (left panel) and protein (right panel) levels. However, blocking STAT3 might cut this effect. (B) Upon the stimulation of IL-10 at indicated concentration for 24 hours, the total (STAT3) and phosphorylated (P-STAT3) levels was measured by Western blot. (C) Following treatment with IL-10 (10ng/ml) for the indicated time points, the total (STAT3) and phosphorylated (P-STAT3) levels was measure by Western blot. (D) The graphical illustration for the binding of STAT3 to the promoter of SEMA4A. (E) ChIP was performed in 22RV1 to detect the enrollment of STAT3 to the promoter of SEMA4A following IL-10 stimulation.

In view of the cooperation between IL-10 and SEMA4A, we wonder whether IL-10, in turn, could regulate SEMA4A expression in PCa cells. Of note, treatment of VCaP cells with recombine human IL-10 up-regulated SEMA4A expression steadily in a concentration-dependent manner (Figure 6A). As STAT3 signaling is established to be activated by IL-10 treatment^18^, we treated 22RV1 cells with IL-10, and the results showed that the levels of p-STAT3 increased in a dose- and time-dependent manner (Figure 6B and C). To further validate how STAT3 regulates SEMA4A, The JARSPAR database was utilized to screen if any binding sites exited in the promoter of SEMA4A and, fortunately, the region encompassing -3243 bp to -3232 bp was found (Figure 6D). Then, ChIP assay was performed, and in contrast to the negative control of IP samples with IgG antibody, specific binding was detected in this promoter region (Figure 6E). Interestingly, more enrichment was observed when the cells were treated with IL-10. The above results further supported the important involvement of SEMA4A, as the direct target of STAT3, during IL-10 functions in the tumor microenvironment.

Base on the above data, we further speculated that STAT3 signaling may be pivotal for SEMA4A and IL-10 to function. We then examined the effects of inhibiting STAT3 signaling on the biological activity of SEMA4A and IL-10. The transwell assay in Figure 5C showed that, after the treatment of stattic, a selective STAT3 inhibitor, the migration ability of 22RV1 cells was inhibited. Similar results were also obtained in co-culture system (Figure 5E), which demonstrated that stattic treatment could attenuate the migration of 22RV1 cells in the presence of rhSEMA4A and IL-10, alone or together. Notably, the *in vivo* experiment showed that the migration of VCaP co-cultured with WPMY-1 was almost blocked when the activity of STAT3 signaling was inhibited by static treatment, in the presence or absence of rhSEMA4A (Figure 4A). Overall, these results suggest that SEMA4A and IL10 may modulate the migration of PCa cells in an STAT3 signaling dependent manner.

## Discussion

Multiple evidences support the notion that semaphorins mediate many of the microenvironmental changes that lead to tumor progression^19^. This may be also the case for PCa, where abnormal expression of Sema3A and Sema4F has been reported to be associated with disease development^12^. Here we show that SEMA4A expression is also up-regulated in PCa than adjacent normal tissues and BPH, which was consistent with the data analysis collecting from previous studies, suggesting its likely involvement in the pathological progression of PCa.

Most deaths related to PCa are due to invasion and metastasis, which are complex processes that required various functional molecules. In this study, we have pinpointed SEMA4A as a novel modulator of tumor cell migration and metastasis both *in vivo* and *in vitro*. Of note, our data suggest that SEMA4A in this soluble form could also dispose PCa cells of a more aggressive phenotype. All these support that SEMA4A may act as a tumor promoter in PCa and it could exacerbate the tumor phenotype and promote metastasis of PCa cells.

Tumor progressed depend on complex interactions between cancer epithelial cells and the surrounding stromal compartment within the tumor microenvironment. The stromal compartment is comprised of multiple nonmalignant cells. Of them, Myofibroblasts present in cancer-activated stromal tissue are the source of many of the signals that direct tumor cell proliferation and metastasis. To further elucidate the role of SEMA4A in PCa, protein array was applied to detect the expression profile of WPMY-1, which was a kind of myofibroblasts, after rhSEMA4A stimulation and IL-10 exhibited the most obvious induction, which was further confirmed by analyzing its secretion in the supernatant of WPMY-1. Previous studies reported that IL-10 could protect against inflammatory via binding to its cognate receptor, IL-10R^20^. Interestingly, our study found that IL-10 could promote the migration and EMT of PCa cells, which was in line with previous reports^17^. More importantly, our study also observed that IL-10 could positively regulate SEMA4A expression, suggesting the existence of a positive loop between them in the milieu to promote PCa progression. We confirmed that the above loop by employing co-cultures of PCa cells and WPMY-1 to measuring the* in vitro* and* in vivo* metastatic phenotypes (migration and lung metastasis), suggesting that SEMA4A and IL-10 might partially mediate the interaction between cancer and stromal cells in the microenvironment of PCa.

Taken together, this study support SEMA4A, especially in this soluble form, could promote PCa progression. More importantly, SEMA4A-IL-10 axis was firstly identified to mediate the crosstalk between PCa cells and stromal cells to promote disease progression. Blocking SEMA4A-IL-10 represents therapeutic potential in PCa.

## Supplementary Material

Supplementary table.

## Figures and Tables

**Table 1:** Relationship between SEMA4A expression level and clinicopathological variables in PCa patients

Variable	SEMA4A levels		*P* value
	Negative and weak (n/%)	Moderate and strong (n/%)	
Age(years)			
≤65	13 (68)	6 (32)	0.943
>65	50 (68)	24 (32)	
Pre PSA (ng ⁄ ml)			
<4	19 (68)	9 (32)	<0.001*
4-10	12 (67)	6 (33)	
>10	32 (68)	15 (32)	
Gleason score			
<7	18 (78)	5 (22)	<0.001*
7	21 (66)	11 (34)	
>7	24 (63)	14 (37)	
Distant Metastasis			
Yes	5 (42)	11 (58)	0.035*
No	58 (72)	19 (28)	
Pathological tumor stage			
≤pT2	47 (72)	18 (28)	0.151
≥pT3	16 (57)	12 (43)	
			

Values not available for all 96 cases.
