# Supplementary material for: SEMA4A promotes prostate cancer invasion: involvement of tumor microenvironment
Source: J Cancer. 2023 Aug 21;14(14):2633–43. doi: 10.7150/jca.86739 (PMC10539395; doi:10.7150/jca.86739)
Supplement: Supplementary file 1 — Supplementary table. [file jcav14p2633s1.pdf]

Supplementary Table 1 Primers used in this study

Gene	Forward	Reverse
SEMA4A	TGGATGGGATGCTCTATTCTGG	GCGGAGGAAGTTGTCGGTC
GAPDH	AATGGGCAGCCGTTAGGAAA	GCGCCCAATACGACCAAATC
